# The Preoperative Assessment and Optimization of Patients Undergoing Major Urological Surgery

**DOI:** 10.1007/s11934-017-0701-z

**Published:** 2017-06-06

**Authors:** Helen W. Cui, Benjamin W. Turney, John Griffiths

**Affiliations:** 1 Department of Urology, Oxford University Hospitals NHS Foundation Trust, Churchill Hospital, Old Road, Oxford, UK; 2 Nuffield Department of Anaesthetics, University of Oxford, Level 6, West Wing, John Radcliffe Hospital, Oxford, OX3 9DU UK

**Keywords:** Preoperative assessment, Urological surgery, CPET, Cystectomy, Nephrectomy, Nephroureterectomy

## Abstract

**Purpose of Review:**

Improving patient outcomes from major urological surgery requires not only advancement in surgical technique and technology, but also the practice of patient-centered, multidisciplinary, and integrated medical care of these patients from the moment of contemplation of surgery until full recovery. This review examines the evidence for recent developments in preoperative assessment and optimization that is of relevance to major urological surgery.

**Recent Findings:**

Current perioperative medicine recommendations aim to improve the short-term safety and long-term effectiveness of surgical treatments by the delivery of multidisciplinary integrated medical care. New strategies to deliver this aim include preoperative risk stratification using a frailty index and cardiopulmonary exercise testing for patients undergoing intra-abdominal surgery (including radical cystectomy), preoperative management of iron deficiency and anemia, and preoperative exercise intervention.

**Summary:**

Proof of the utility and validity for improving surgical outcomes through advances in preoperative care is still evolving. Evidence-based developments in this field are likely to benefit patients undergoing major urological surgery, but further research targeted at high-risk patients undergoing specific urological operations is required.

## Introduction

The number of patients undergoing major urological surgery is growing. From 2011 to 2015, there has been an increase in the number of nephrectomies, radical cystectomies, and radical prostatectomies being performed in the UK by 9.5, 8.5, and 53% respectively [1–5]. Improvements in surgical technique and technology [6], as well as intraoperative and postoperative care, [7] and enhanced recovery programs [8], have further reduced patient morbidity and mortality from major urological surgery.

However, the current and future patient populations undergoing major urological surgery is increasingly older with a greater number of associated medical comorbidities [9, 10]. A significant proportion of these patients therefore carry a greater risk of experiencing increased peri- and postoperative morbidity and mortality even before “knife-to-skin” occurs. Across the surgical specialties, there is increased recognition of the importance of preoperative assessment and perioperative medical care within the surgical pathway to evaluate and manage the patient above and beyond operation-specific issues [11]. A recent National Confidential Enquiry into Patient Outcome and Death (NCEPOD) report into perioperative care of surgical patients in the UK found that although “high-risk patients” only comprise 10% of the overall inpatient surgical workload, they account for 80% of deaths after surgery [12]. A key finding of the NCEPOD report was that preoperative assessment and intervention pathways play a vital role in both identifying and optimizing those patients at higher risk of morbidity and mortality after surgery [12].

Although the concept of preoperative medicine and its role in improving surgical outcomes is gaining wide recognition within the anesthetic literature and associated governing bodies [13–15], there is currently a relatively unmet need for similar recognition within the surgical literature and surgical governing bodies. A key recommendation of the Royal College of Anaesthetists Perioperative Medicine Vision Document is the importance of a true multidisciplinary perioperative team, with surgeons at the heart of it [13]. In the era of surgeon-specific outcome reporting, the role of the surgeon and close working with anesthetic colleagues is more important than ever, not just during the operation, but also in the pre- and postoperative period [16].

This review will consider the literature investigating the recent recommendations and developments in the field of preoperative assessment and preoperative interventions, and how this has been, or can be, applied to major urological surgery. Table 1 summarizes the main studies highlighted in this review.

**Table 1:** Summary table of available evidence for recent developments in preoperative assessment and intervention for specific major urological operations where studies exist, or for major abdominal surgery which have included unspecified urological operations. Levels of evidence based on the “Oxford Centre for Evidence-Based Medicine 2009 Levels of Evidence” [17]

Preoperative assessment method or intervention	Level of evidence	Author(s) year	Operations included	Outcome(s) measured	Conclusion
ASA grade for preoperative patient risk stratification	IIb	Djaladat et al. 2014 [75•]	Radical cystectomy	Overall survival, 90-day complication rate	A high ASA grade of ≥3 was associated with decreased overall survival and increased 90-day complication rate
	IIb	Malavaud et al. 2001 [76•]	Radical cystectomy	30-day morbidity	A high ASA grade of ≥3 was associated with increased major complications within 30 days postoperatively
CPET for preoperative patient risk stratification	Ib	Tolchard et al. 2015 [43•]	Radical cystectomy	Postoperative complications, length of stay	CPET parameters AT, VE/VCO_2_, and hypertension as risk factors predictive of 90-day complication rate
	Ib	Prentis et al. 2013 [42•]	Radical cystectomy	Postoperative complications, length of stay, mortality	AT is a significant predictor for major postoperative complications (Clavien-Dindo grade ≥3) and length of stay
Preoperative fragility assessment	IIb	Lascano et al. 2015 [46•]	Radical cystectomy, prostatectomy, nephrectomy, nephroureterectomy	Mortality, incidence of Clavien-Dindo IV complications	Patients with increased fragility had a higher incidence of 30-day postoperative mortality and Clavien-Dindo IV complications but this was not superior to using ASA grade
Treatment of preoperative iron-deficiency anemia with iron supplementation	Ib	Froessler et al. (2016) [18]	Abdominal surgery	Allogeneic blood transfusion, change in Hb level, length of stay	Patients treated with intravenous iron had 60% reduction in allogeneic blood transfusion events, higher Hb level by day of admission, shorter length of stay and higher Hb level at 4 weeks after discharge compared with the control group receiving usual care
Preoperative exercise intervention or “prehabilitation”	Ia	Hijazi et al. 2017 [66•]	Intra-abdominal cancer operations	Performance on 6MWT or CPET (AT and peak VO_2_)	Studies too heterogeneous, lack of evidence to support prehabilitation
	Ib	Jensen et al. 2015 [68]	Radical cystectomy	Length of stay, severity of complications	No difference in length or stay or severity of complications between prehabilitation and control group

*ASA* American Association of Anesthesiologists physical status classification, *CPET* cardiopulmonary exercise test, *AT* anaeorbic threshold, *VO*_*2*_ *max* maximum oxygen consumption, *VE/VCO*_*2*_ ventilatory equivalent for carbon dioxide, *CSHA-FI* Canadian Study of Health and Aging fragility index, *GDT* goal-directed therapy, *6MWT* 6 min walk test

## Preoperative Assessment

A key aim of preoperative assessment is to use validated scoring systems and risk indices to identify patients at predicted higher risk of complications from surgery. Examples are the NCEPOD Surgical Outcome Risk Tool (SORT) [25, 26], The American College of Surgeons Mortality and Morbidity Risk Calculator [27], the P-POSSUM [28, 29], and the Lee’s Cardiac Risk Index [30]. This can then guide not only the planning of surgery and perioperative care interventions, but also allow informed decision making with the patient, such that, in some cases, undertaking surgery may not be the best option. This is especially pertinent for those patients with prostate and bladder cancer that have the option of radiotherapy. Until recent times, this was largely determined by the surgeon’s clinical acumen in being able to assess risk based on the “look” of a patient. However, current theories on how surgery impacts on patient physiology provide a more scientific approach for how patient factors can influence surgical outcome. Where higher-risk patients have been identified, the available time ahead of surgery can then be used to optimize and treat any relevant existing comorbidities and to make a detailed peri- and postoperative medical management plan for them [13].

### Preoperative Tests

There is considerable variation in the preoperative assessment process between countries, and the surgical centers within countries [12]. There are numerous preoperative patient risk assessment indices that include subjective and objective questionnaires, scoring systems, and static and dynamic tests. However, to date, there is limited consensus on the efficacy or cost efficiency of these assessments [11]. Current guidelines from both the American Urological Association (AUA) and the European Association of Urology (EAU) provide few recommendations on the general preoperative assessment for major urological surgery. The AUA guidelines only offer recommendations with respect to anticoagulation management [31], and the EAU guidelines include an acknowledgement that the American Association of Anesthesiologists (ASA) grade can predict risk of major complications following radical cystectomy [32].

**Fig. 1:**
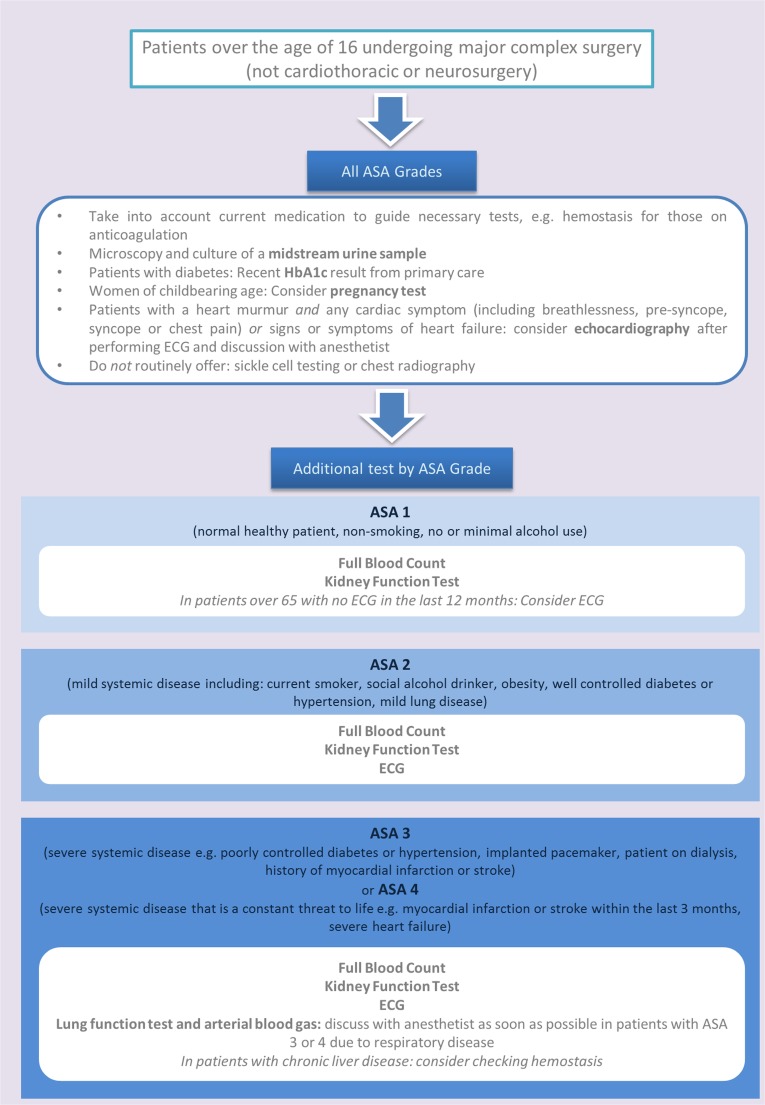
Flowchart of preoperative test selection relevant to major urological surgery based on current recommendations from the NICE [14]

In the absence of surgical specialty-specific guidelines, the National Institute of Health and Care Excellence (NICE) [14] in the UK and the American Colleges of Cardiology, Radiology and Anaesthesiologists [33] offer general guidelines for preoperative testing, with consensus between their recommendations. Figure 1 offers a selected summary of these guidelines applicable to major urological surgery. Important principles are the need to fully investigate high-risk patients in preoperative assessment clinics [12]; the need to avoid excessive preoperative testing with the potential for spurious results, patient anxiety, delay to surgery and increased cost [34, 35]; the need to review any results available from primary care to highlight issues and avoid unnecessary repetition [14]; and the need to follow specialized recommendations for patients with obesity and diabetes due to the increased risk of complications for these patient groups [36].

### Cardiopulmonary Exercise Testing

Preoperative cardiopulmonary exercise testing (CPET) has been used for many years to quantify the exercise capacity of the patient [37]. Surgery is widely recognized as a physiological insult that results in a systemic inflammatory response syndrome (SIRS) [21]. Thus, the result of surgical insult is a globally increased tissue demand for oxygen delivery combined with a reduced ability for these tissues to extract oxygen, both during the inflammatory insult (“the surgery”) and afterwards (“the recovery”) [20]. This response can result in tissue oxygenation debt during recovery from the surgery phase, with subsequent adverse consequences on end-organ function and wound healing [38]. A surgical patient’s exercise capacity tested during CPET reflects their ability to increase their cardiac output, and therefore oxygen delivery, to sufficiently meet the increased metabolic need of the postoperative phase, and this ability has been associated with improved survival after major surgery [39]. CPET has been shown to be able to identify those patients with a reduced capacity to cope with this increased oxygen demand and therefore identify the high-risk patient group for intraoperative and postoperative complications and delayed recovery from surgery [39, 40]. Decisions can then be made before surgery as to potential preoperative interventions to try and ameliorate this risk and to the level of care required postoperatively, for example, the need for high dependency (level 2) or intensive care (level 3) resources [12].

The largest preoperative CPET evidence base is currently in cardiothoracic surgery, but there is a growing interest for its utility in patients undergoing intra-abdominal surgery [22]. A recent NCEPOD report has shown that preoperative CPET is routinely undertaken in approximately 40% of UK hospitals as part of preoperative assessment of patients undergoing major surgery [13]. During a CPET, the patient is asked to exercise to their maximal effort on a treadmill or against increasing resistance on a static exercise bike, with continuous ECG, blood pressure, oxygen saturation, and inspiratory and expiratory ventilatory gas monitoring. The exercise component of the test takes up to 15 min, and combined with a full anesthetic consultation, a CPET clinic routinely lasts up to 1 h.

CPET generates various parameters that characterize the patient’s cardiorespiratory reserve and some of these parameters have been shown to prognosticate for postoperative mortality and morbidity across a range of operations. The two main CPET parameters cited in the current literature are the peak VO_2_ and the anaerobic threshold (AT) [41]. The peak VO_2_ is the maximum level of oxygen consumption the patient can achieve at maximal effort; the AT is the threshold of oxygen consumption at which aerobic respiration is supplemented by anaerobic respiration in order to eliminate excess carbon dioxide. Both parameters are expressed in milliliter/kilogram/minute.

A review of the potential utility of CPET in identifying high-risk patients before intra-abdominal surgery has been recently published [22]. Heterogeneity of the data of the included 37 prognostic studies precluded a meta-analysis. A quantitative synthesis of the 10 studies which included patients undergoing a mixture of major abdominal surgeries (including 2 studies solely involving radical cystectomy patients) showed 4 studies reporting an AT of <11 ml/kg/min to be a significant predictor of postoperative mortality. However, one study showed AT was not a significant predictor of mortality, and of the two studies of CPET before radical cystectomy, one had an insufficient number of postoperative deaths and no statistical analysis for mortality [42•], and the other had an insufficient sample size [43•]. In terms of predicting postoperative morbidity, five studies of intra-abdominal surgery, including the two studies of radical cystectomy patients only, showed AT was again the strongest predictor. Of the radical cystectomy studies, Prentis and colleagues showed an AT <12 ml/kg/min to be predictive of in-hospital morbidity [42•], and Tolchard and colleagues showed a significantly different AT and peak VO_2_ between patients who experienced a Clavien-Dindo grade ≥2 complication within 90 days postoperatively and those who did not [43•]. This systematic review suggests that different CPET parameters will have a varied relationship and predictive ability for different surgical interventions and clinical outcome measures. At the current time, there is no single threshold of one CPET parameter that can be used to stratify risk for surgery [22]. Further studies with adequate a priori sample size calculation and robust recording of standardized outcomes measures are therefore required to determine the true utility of CPET in predicting outcome from specific, major urological operations [22]. In addition to the value of the objective measures obtained during CPET, it is likely that the anesthetic review usually conducted as part of CPET clinic, which will include a review of comorbidities, medications, and formulation of a bespoke preoperative optimization plan contributes to the utility of CPET as a risk stratification tool.

### Frailty Assessment

As well as formal cardiorespiratory assessment, there is growing evidence for the concept of patient “frailty” as an important risk factor for postoperative complications in the elderly population [44], which offers more information than using age alone. The Royal College of Anaesthetists in the UK recommends that extension of the multidisciplinary team to involve elderly care physicians should be considered for the preoperative assessment and management of patients aged 70 and over [13]. Frailty assessment takes into account an elderly patient’s strength, energy, cognition, health status, speed of any functional decline, and the impact on their activities of daily living—leading to the concept of patient “vulnerability” [45]. The Canadian Study of Health and Aging Frailty Index (CSHA-FI) has been validated in the general elderly population to predict risk of death and institutionalization [45]. An abbreviated version of this score has been validated as a preoperative risk stratification tool across different surgical specialties [46•].

In 2005–2013, a large retrospective analysis of the outcomes of 41,681 patients that had undergone major urological cancer surgery was published [46•]. Urological procedures included prostatectomy, cystectomy, nephrectomy, and nephroureterectomy, and variables studied were a modified frailty index score consisting of 11 variables from the CSHA-FI, history of metastasis, chemotherapy or radiation exposure, weight loss, and renal failure. This retrospective analysis showed patients with a high modified frailty index score had a significantly higher odds of a Clavien-Dindo 4 event (OR = 3.70, 95% CI 2.87 to 4.79) and 30-day mortality (OR = 5.95, 95% CI 3.72 to 9.51) compared to non-frail patients [46•]. Although this modified frailty index was superior to the Charlson Comorbidity Index in predicting 30-day mortality and Clavien-Dindo 4 events, it was still not superior to using the ASA grade. As with other prognostic indicators that lack propensity adjustment, heterogeneity of both the operative intervention and the patient characteristics will affect the strength of the conclusions of how risk factors affect clinical outcomes. The heterogeneity of major urological operations included in this review may have affected the prognostic ability of the modified frailty index. For example, cystectomy patients had a 30-day mortality rate of 2.6% and Clavien-Dindo 4 complication rate of 9.5%, whereas prostatectomy patients, as can be expected, had a lower 30-day mortality rate of only 0.2% and Clavien-Dindo 4complication rate of 1.1%. A separate analysis of 2679 cystectomy patients from the same database showed a similar predictive ability for the modified frailty index score to predict postoperative complications [24]. Two limitations of this analysis of cystectomy patients are the heterogeneity with respect to histological staging, exposure to neoadjuvant chemotherapy and type of urinary diversion performed, and the fact that the utility of the modified frailty index score was not compared to other validated risk stratification tools.

Although systematic preoperative frailty assessment of elderly patients has face validity, the additional utility of any individual frailty scoring system in predicting outcomes from specific urological operations needs further evaluation. Specifically, which components of a frailty scoring system can add value for mortality and morbidity risk stratification, over and above the stated ASA grade is unclear. Moreover, given the importance to the patient of longer-term health-related quality of life outcomes, such as functional outcome, future studies of prognostic indices need to incorporate these outcomes measures in addition to patient mortality and morbidity.

## Preoperative Intervention

Established preoperative interventions in major urological surgery to try and improve cardiorespiratory reserve, and thus lessen surgical risk, include: optimization of patient comorbidities and related medications, optimization of nutritional status, and cessation of smoking [8]. New developments in preoperative intervention and optimization include evidence for the identification and treatment of anemia, and optimization of physiological reserve through preoperative exercise intervention.

### Preoperative Iron Deficiency and Anemia

Patients scheduled for major urological cancer surgery are likely to have iron deficiency, with or without anemia. Iron deficiency is associated with approximately 43% of all malignancies [47], and the reported prevalence of preoperative anemia for patients undergoing radical prostatectomy is 8% [23], radical nephrectomy is 35% [48], nephroureterectomy is 39.7% [49], and radical cystectomy is 45% [50]. As part of the concept of better ‘Patient Blood Management’, is the growing evidence for early management of preoperative anemia using a multimodal and individualized approach, including treatment of preoperative anemia with iron supplementation, leading to a significant reduction of the need for perioperative allogeneic transfusion [18, 19, 47].

The significance of iron deficiency, and anemia, as a preoperative risk factor is supported by evidence that such patients have a higher rate of perioperative transfusion of blood products [51]. Allogeneic blood transfusion has immunosuppressive effects and, transfusion of a single unit of packed red blood cells has been associated with increased postoperative morbidity and mortality [52]. This detrimental effect of perioperative blood transfusion has also been demonstrated for patients undergoing radical cystectomy and prostatectomy [53, 54].

In patients undergoing major, non-cardiac surgery, preoperative anemia has recently been shown to be an independent risk factor for postoperative mortality and morbidity, in addition to the increased risk of need for transfusion [55, 56]. In one study of 684 patients undergoing radical cystectomy, preoperative anemia was found to be an independent predictor of disease recurrence, cancer-specific mortality, and all-cause mortality [57]. Of note, this study showed that although perioperative blood transfusion significantly increased all-cause mortality independent of preoperative anemia, those with preoperative anemia who received perioperative blood transfusion had no significant difference in outcome measures. The authors suggest that correction of preoperative anemia may have a survival benefit irrespective of reducing transfusion rate [57]. Preoperative anemia has also been found to be an independent predictor of cancer-specific mortality for patients undergoing radical nephrectomy [48] and nephroureterectomy [49]*.* These studies raise the possibility that the presence of preoperative anemia could be an important risk stratification tool for major urological surgery [48, 49, 57]. However, once the effect of its association with disease severity and other patient risk factors are accounted for, preoperative anemia in itself, is a relatively weak independent risk factor for poor surgical outcome [58].

As patients undergoing radical and partial nephrectomy, and radical cystectomy, have reported perioperative allogeneic transfusion rates of between 5–11% [59] and 20–30% [1, 60], respectively, targeting the preoperative management of anemia in these patients is likely to be of benefit. Intraoperative cell-salvage therapy during radical cystectomy has been shown to reduce the requirement for allogeneic transfusion [61], but no nephrectomy- or cystectomy-specific studies have been conducted regarding the preoperative management of anemia, and thus no recommendations have been made regarding preoperative anemia in a recent systematic review of enhanced recovery for urological surgery [8, 62].

In the absence of urology-specific guidelines, generic guidance can be taken from the 2017 “International Consensus Statement on the Perioperative Management of Anaemia and Iron Deficiency” [47]. This states that, for patients undergoing major surgery, where estimated blood loss is >500 ml, treatment of iron deficiency with, or without anemia, is recommended. Treatment should be with oral iron supplementation (or intravenous iron supplementation for those unable to tolerate oral or who have <6 weeks before surgery) to achieve a Hb of >13 g/dl in both sexes, with the primary aim to reduce transfusion rate and thereby improve outcomes from surgery [18, 47, 63]. However, to date, no Level 1 evidence exists as to whether improving Hb levels preoperatively, can significantly impact the postoperative morbidity and mortality rates beyond that which is associated with the increased risk of transfusion [47]. Results are awaited from an ongoing, multi-center, UK RCT on the effectiveness and cost-effectiveness of preoperative intravenous iron supplementation in reducing transfusion rate, length of stay, and postoperative complication rate [64].

### Preoperative Exercise Intervention

It is hypothesized that better physical fitness preoperatively improves a patient’s ability to meet the increased oxygen demand during and after surgery. Preoperative physical activity or exercise has been shown to improve a patient’s peri- and postoperative ability to extract oxygen and tolerate the ischaemic conditions of surgery, which lessens the impact of any deficit in oxygen delivery [38]. Randomized controlled trials (RCTs) have been conducted in a variety of surgical populations to investigate the efficacy of preoperative exercise intervention as a form of preoperative rehabilitation or “prehabilitation” [65, 66•, 67]. There is now embryonic evidence for “prehabilitation” in patients undergoing major urological surgery in the form of one completed RCT [68].

Jensen and colleagues conducted a RCT of preoperative exercise intervention in 107 patients undergoing radical cystectomy [68]. In addition to standard care, the intervention group received 2 weeks of preoperative training involving a twice daily home exercise program on a step trainer (provided by the hospital to the patient’s home) and six different muscle strength and endurance exercises. The intervention also comprised of a postoperative phase of 1 week of in-hospital exercises and mobilization supervised by a physiotherapist. The standard of care was based on existing Enhanced Recovery After Surgery (ERAS) principles. Both the control and intervention groups received either robotic-assisted radical cystectomy or a minilaparotomy and urinary diversion based preoperatively on patient characteristics and preference. Compliance to the prehabilitation program (defined as completion of at least 75% of the program) was found to be 59% and was checked by a phone call after 1 week. There was no significant difference in both length of stay as the primary outcome, or severity of complications as the secondary outcome, between the intervention and control groups [68]. It is postulated that the lack of effect shown by the prehabilitation intervention could be due to a number of factors that include too short a preoperative intervention time of 2 weeks; the use of less invasive methods of radical cystectomy and good application of ERAS principles that delivered a general reduction in length of stay and complication severity; and difficulties of investigator supervision to the home exercise program with possible poor overall patient compliance [68].

The most recent systematic review of prehabilitation in intra-abdominal cancer surgery was published earlier this year by Hijazi and colleagues, and includes the study of radical cystectomy patients by Jensen and colleagues [66•]. This systematic review undertook qualitative synthesis of seven RCTs and two prospective, non-randomized trials. In line with previously published systematic reviews [69–71] in this area, Hijazi and colleagues concluded that, at the current time, there is limited evidence that preoperative prehabilitation leads to a clinically significant physiologic improvement in patients undergoing major elective abdominal surgery [66•]. The lack of significant clinical effect from prehabilitation is likely to be due to a variety of factors that include lack of adherence to the individual exercise program, uncertainty and variation as to the ideal physiologic endpoint to measure, uncertainty as to the gold-standard components of a prehabilitation program, uncertainty as to the optimal preoperative duration of the prehabilitation program, and uncertainty as to which groups of patients and/or surgical procedures (e.g., open versus laparoscopic) would benefit most from a prehabilitation program [66•, 69].

A major criticism of existing prehabilitation trials is the lack of patient risk stratification in selection for inclusion into an individual trial [69]. A variety of validated tools are available that can predict those patients at higher risk of experiencing poorer surgical outcomes [11, 12]. As patients predicted to be at higher risk are most likely to benefit from prehabilitation, a more rigorous approach would be to use a validated screening tool to risk stratify higher-risk patients into prehabilitation programs.

A major challenge in conducting studies of preoperative interventions remains the ability to demonstrate a significant difference in important clinical outcomes such as length of stay, major postoperative complications, and mortality. To date, studies are often not adequately powered to detect significant differences in these clinical outcomes, given that the rates of major complications and mortality remain low. When faced with a comorbid elderly population undergoing a range of intra-abdominal operations, both open and laparoscopic, benign and oncological, there is currently no single preoperative intervention that has been shown to lead to an improvement in both clinical outcomes and quality of life outcomes [72]. Hence, the approach needs to be multidisciplinary and multimodal [68, 73]. Future trials of separate individual components of prehabilitation are unlikely to show clear benefits in clinical outcomes, and the future direction of research in this area should be based on the concept of implementing a perioperative care pathway that is an aggregation of marginal gains [13, 74].

## Conclusion

Few would argue against the fact that optimal, evidence-based, coordinated, preoperative assessment and intervention leads to improved outcomes from surgery. The challenge is being able to demonstrate the improvement in research studies, particularly randomized controlled trials. High-level evidence supporting the use of certain preoperative assessment tests and interventions is difficult to generate—due in part to the complex nature of the surgical process itself. To date, novel preoperative processes have been investigated and implemented, but despite having good face validity, clear effects of these processes on important clinical outcomes remain limited. However, the evidence base for how to further reduce surgical risk, and improve outcomes from surgery through preoperative assessment and intervention, is still in its infancy. For patients undergoing major urological surgery, it remains possible that accurate risk stratification through the use of preoperative CPET and frailty indices, in addition to an evidence-based approach to prehabilitation and the management of preoperative anemia, will be shown to lead to improved, clinically significant surgical outcomes.

## Abbreviations

ACC: American College of Cardiologists
ACS NSQIP: American College of Surgeons’ National Surgical Quality Improvement Program
AHA: American Heart Association
ASA: American Association of Anaesthesiologists physical status classification
AT: Anaerobic threshold (also known as lactate threshold)
AUA: American Urology Association
CI: Confidence interval
CPET: Cardiopulmonary Exercise Test
CSHA-FI: Canadian Study of Health and Aging Frailty Index
EAU: European Association of Urology
ERAS: Enhanced Recovery After Surgery
ESA: European Society of Anaesthesiologists
ESC: European Society of Cardiologists
NCEPOD: National Confidential Enquiry into Patient Outcome and Death
NICE: National Institute of Health and Care Excellence
PWC-170: Physical Work Capacity cycle test
RCT: Randomized Controlled Trial
SIRS: Systemic Inflammatory Response Syndrome
SORT: Surgical Outcome Risk Tool
UK: United Kingdom
VE/VCO_2_: Ventilatory equivalent for carbon dioxide
VO_2_: Oxygen consumption
6MWT: 6 Min Walk Test
